# Supplementary material for: Association of frequency of television watching with overweight and obesity among women of reproductive age in India: Evidence from a nationally representative study
Source: PLoS One. 2019 Aug 29;14(8):e0221758. doi: 10.1371/journal.pone.0221758 (PMC6715273; doi:10.1371/journal.pone.0221758)
Supplement: S2 File — (DOCX) [file pone.0221758.s002.docx]

Supplementary Tables

**Table A: Crude and Adjusted odds ratios for factors associated with overweight and obesity compared to normal weight among women of reproductive age group in India, NFHS 2015-16.**

| **Variable** | **Model 1 (COR)** | | | **Model 2 (AOR)** | | |
| --- | --- | --- | --- | --- | --- | --- |
|  | OR | Lower Limit | Upper Limit | OR | Lower Limit | Upper Limit |
| **Age Group (in years)** |  |  |  |  |  |  |
| 15-24 | Ref |  |  | Ref |  |  |
| 25-34 | 3.58*** | 3.52 | 3.63 | 2.37*** | 2.32 | 2.42 |
| 35-49 | 6.33*** | 6.24 | 6.43 | 4.18*** | 4.09 | 4.28 |
| **Place of Residence** |  |  |  |  |  |  |
| Rural | Ref |  |  | Ref |  |  |
| Urban | 2.56*** | 2.51 | 2.62 | 1.42*** | 1.39 | 1.44 |
| **State of Residence** |  |  |  |  |  |  |
| Andaman and Nicobar islands | Ref |  |  | Ref |  |  |
| AndhraPradesh | 1.10 | 0.93 | 1.29 | 1.04 | 0.91 | 1.19 |
| Arunachal Pradesh | 0.72*** | 0.61 | 0.84 | 1.03 | 0.90 | 1.17 |
| Assam | 0.37*** | 0.31 | 0.43 | 0.60*** | 0.53 | 0.68 |
| Bihar | 0.28*** | 0.24 | 0.33 | 0.63*** | 0.56 | 0.71 |
| Chandigarh | 1.91*** | 1.41 | 2.59 | 1.03 | 0.80 | 1.31 |
| Chhattisgarh | 0.29*** | 0.25 | 0.34 | 0.42*** | 0.37 | 0.48 |
| Dadra and Nagar Haveli | 0.48*** | 0.35 | 0.66 | 0.53*** | 0.41 | 0.69 |
| Daman and Diu | 1.05 | 0.83 | 1.33 | 0.79* | 0.65 | 0.96 |
| Goa | 1.19^1^ | 0.95 | 1.50 | 0.78** | 0.65 | 0.94 |
| Gujarat | 0.57*** | 0.48 | 0.66 | 0.57*** | 0.50 | 0.65 |
| Haryana | 0.67*** | 0.57 | 0.78 | 0.50*** | 0.44 | 0.57 |
| Himachal Pradesh | 0.92 | 0.78 | 1.08 | 0.82** | 0.72 | 0.93 |
| Jammu and Kashmir | 0.85* | 0.73 | 0.99 | 1.24** | 1.09 | 1.40 |
| Jharkhand | 0.23*** | 0.20 | 0.27 | 0.43*** | 0.38 | 0.48 |
| Karnataka | 0.62*** | 0.53 | 0.72 | 0.65*** | 0.58 | 0.74 |
| Kerala | 1.22* | 1.04 | 1.43 | 0.78*** | 0.68 | 0.89 |
| Lakshadweep | 1.80*** | 1.35 | 2.41 | 1.13 | 0.89 | 1.42 |
| MadhyaPradesh | 0.34*** | 0.29 | 0.39 | 0.46*** | 0.41 | 0.52 |
| Maharashtra | 0.55*** | 0.47 | 0.64 | 0.54*** | 0.47 | 0.60 |
| Manipur | 0.85* | 0.72 | 1.00 | 1.16* | 1.02 | 1.32 |
| Meghalaya | 0.38*** | 0.32 | 0.45 | 0.57*** | 0.50 | 0.65 |
| Mizoram | 0.59*** | 0.50 | 0.69 | 0.52*** | 0.46 | 0.59 |
| Nagaland | 0.46*** | 0.39 | 0.54 | 0.61*** | 0.53 | 0.70 |
| Delhi | 1.39*** | 1.17 | 1.66 | 0.79** | 0.69 | 0.91 |
| Odisha | 0.41*** | 0.35 | 0.48 | 0.68*** | 0.60 | 0.77 |
| Puducherry | 1.51*** | 1.24 | 1.82 | 0.97 | 0.83 | 1.13 |
| Punjab | 1.23* | 1.05 | 1.43 | 0.85* | 0.75 | 0.96 |
| Rajasthan | 0.37*** | 0.32 | 0.43 | 0.46*** | 0.41 | 0.52 |
| Sikkim | 1.05 | 0.88 | 1.26 | 1.15 | 1.00 | 1.33 |
| Tamil Nadu | 1.04 | 0.90 | 1.22 | 0.92 | 0.81 | 1.03 |
| Tripura | 0.50*** | 0.41 | 0.60 | 0.64*** | 0.55 | 0.74 |
| UttarPradesh | 0.44*** | 0.38 | 0.51 | 0.71*** | 0.63 | 0.80 |
| Uttarakhand | 0.55*** | 0.47 | 0.64 | 0.57*** | 0.50 | 0.65 |
| West Bengal | 0.52*** | 0.45 | 0.61 | 0.74*** | 0.65 | 0.84 |
| Telangana | 0.75** | 0.63 | 0.89 | 0.77*** | 0.68 | 0.89 |
| **Highest Educational Status** |  |  |  |  |  |  |
| No Formal Education | Ref |  |  | Ref |  |  |
| Primary | 1.06*** | 1.04 | 1.08 | 1.18*** | 1.15 | 1.20 |
| Secondary | 0.83*** | 0.81 | 0.84 | 1.25*** | 1.23 | 1.28 |
| Higher | 0.89*** | 0.87 | 0.91 | 1.29*** | 1.26 | 1.32 |
| **Wealth index** |  |  |  |  |  |  |
| Poorest | Ref |  |  | Ref |  |  |
| Poorer | 1.77*** | 1.73 | 1.81 | 1.61*** | 1.57 | 1.65 |
| Middle | 2.81*** | 2.74 | 2.87 | 2.37*** | 2.31 | 2.43 |
| Richer | 4.44*** | 4.34 | 4.54 | 3.53*** | 3.44 | 3.63 |
| Richest | 6.68*** | 6.52 | 6.83 | 5.01*** | 4.86 | 5.17 |
| **Marital Status** |  |  |  |  |  |  |
| Single | Ref |  |  | Ref |  |  |
| Married | 4.79*** | 4.72 | 4.87 | 1.98*** | 1.92 | 2.04 |
| Separated/ Divorced/ Widowed | 4.58*** | 4.44 | 4.71 | 1.74*** | 1.67 | 1.81 |
| **Parity** |  |  |  |  |  |  |
| 0 | Ref |  |  | Ref |  |  |
| 1 | 2.89*** | 2.84 | 2.95 | 1.03 | 1.00 | 1.06 |
| 2 | 4.17*** | 4.10 | 4.24 | 1.13*** | 1.10 | 1.16 |
| 3 | 4.31*** | 4.23 | 4.39 | 1.14*** | 1.11 | 1.18 |
| 3+ | 4.15*** | 4.08 | 4.23 | 1.17*** | 1.13 | 1.20 |
| **Number of Household Member** |  |  |  |  |  |  |
| ≤5 | Ref |  |  | Ref |  |  |
| >5 | 0.80*** | 0.80 | 0.81 | 0.91*** | 0.89 | 0.92 |
| **Frequency of watching TV** |  |  |  |  |  |  |
| Not at all | Ref |  |  | Ref |  |  |
| Less than once a week | 1.18*** | 1.15 | 1.22 | 1.05*** | 1.02 | 1.08 |
| At least once a week | 1.47*** | 1.44 | 1.50 | 1.10*** | 1.08 | 1.13 |
| Almost every day | 1.93*** | 1.89 | 1.96 | 1.24*** | 1.21 | 1.26 |

*NFHS: National Family Health Survey*

*CI: Confidence Interval*

*COR: Crude Odds Ratio; AOR: Adjusted Odds Ratio*

*^1^Variable with p-value less than <0.2 from unadjusted model were included into multivariable analysis*

**p-value<0.05, **p-value<0.01, *** p-value<0.001*

**Table B: Crude and Adjusted odds ratios for factors associated with overweight and obesity compared to normal weight among women of reproductive age group in India residing in urban area, NFHS 2015-16.**

| **Variable** | COR | Lower Limit | Upper Limit | AOR | Lower Limit | Upper Limit |
| --- | --- | --- | --- | --- | --- | --- |
| **Age Group (in years)** |  |  |  |  |  |  |
| 15-24 | Ref |  |  | Ref |  |  |
| 25-34 | 3.83*** | 3.73 | 3.93 | 2.38*** | 2.30 | 2.46 |
| 35-49 | 7.66*** | 7.46 | 7.85 | 4.33*** | 4.17 | 4.49 |
| **State of Residence** |  |  |  |  |  |  |
| Andaman and Nicobar islands | Ref |  |  | Ref |  |  |
| Andhra Pradesh | 1.54** | 1.15 | 2.07 | 1.61** | 1.21 | 2.14 |
| Arunachal Pradesh | 0.74* | 0.55 | 0.98 | 0.99 | 0.74 | 1.31 |
| Assam | 0.63*** | 0.47 | 0.84 | 0.76 | 0.57 | 1.01 |
| Bihar | 0.47*** | 0.35 | 0.62 | 0.72* | 0.55 | 0.96 |
| Chandigarh | 1.43* | 1.01 | 2.03 | 1.32 | 0.94 | 1.85 |
| Chhattisgarh | 0.52*** | 0.39 | 0.69 | 0.60*** | 0.45 | 0.79 |
| Dadra and Nagar Haveli | 0.79 | 0.52 | 1.20 | 0.83 | 0.56 | 1.25 |
| Daman and Diu | 0.82 | 0.59 | 1.14 | 0.84 | 0.61 | 1.17 |
| Goa | 1.08 | 0.77 | 1.51 | 0.99 | 0.71 | 1.38 |
| Gujarat | 0.87 | 0.65 | 1.15 | 0.86 | 0.66 | 1.13 |
| Haryana | 0.64** | 0.48 | 0.85 | 0.56*** | 0.42 | 0.73 |
| Himachal Pradesh | 1.08 | 0.77 | 1.53 | 1.00 | 0.71 | 1.40 |
| Jammu and Kashmir | 1.09 | 0.82 | 1.46 | 1.41** | 1.07 | 1.87 |
| Jharkhand | 0.45*** | 0.34 | 0.60 | 0.60*** | 0.46 | 0.79 |
| Karnataka | 0.78^1^ | 0.59 | 1.03 | 0.89 | 0.68 | 1.17 |
| Kerala | 0.97 | 0.73 | 1.29 | 0.81 | 0.61 | 1.07 |
| Lakshadweep | 1.36^1^ | 0.96 | 1.93 | 1.41 | 1.01 | 1.98 |
| Madhya Pradesh | 0.55*** | 0.41 | 0.72 | 0.61*** | 0.47 | 0.80 |
| Maharashtra | 0.76^1^ | 0.58 | 1.01 | 0.79 | 0.60 | 1.03 |
| Manipur | 0.79^1^ | 0.59 | 1.05 | 1.27 | 0.96 | 1.67 |
| Meghalaya | 0.40*** | 0.29 | 0.54 | 0.57*** | 0.42 | 0.77 |
| Mizoram | 0.59*** | 0.44 | 0.78 | 0.63** | 0.48 | 0.83 |
| Nagaland | 0.48*** | 0.36 | 0.64 | 0.67** | 0.50 | 0.89 |
| Delhi | 1.01 | 0.76 | 1.34 | 0.98 | 0.75 | 1.30 |
| Odisha | 0.80^1^ | 0.60 | 1.06 | 1.10 | 0.83 | 1.44 |
| Puducherry | 1.17 | 0.87 | 1.57 | 1.12 | 0.84 | 1.49 |
| Punjab | 1.00 | 0.75 | 1.33 | 0.84 | 0.64 | 1.10 |
| Rajasthan | 0.55*** | 0.42 | 0.73 | 0.57*** | 0.43 | 0.74 |
| Sikkim | 1.02 | 0.74 | 1.40 | 1.25 | 0.92 | 1.70 |
| Tamil Nadu | 1.05 | 0.79 | 1.38 | 1.11 | 0.85 | 1.46 |
| Tripura | 0.62** | 0.45 | 0.85 | 0.77 | 0.57 | 1.05 |
| Uttar Pradesh | 0.64** | 0.49 | 0.84 | 0.83 | 0.63 | 1.08 |
| Uttarakhand | 0.79^1^ | 0.59 | 1.05 | 0.83 | 0.63 | 1.10 |
| West Bengal | 0.82^1^ | 0.62 | 1.09 | 1.08 | 0.82 | 1.43 |
| Telangana | 1.08 | 0.80 | 1.45 | 1.17 | 0.88 | 1.56 |
| **Highest Educational Status** |  |  |  |  |  |  |
| No Formal Education | Ref |  |  | Ref |  |  |
| Primary | 1.02 | 0.98 | 1.06 | 1.15*** | 1.11 | 1.20 |
| Secondary | 0.78*** | 0.76 | 0.80 | 1.20*** | 1.16 | 1.24 |
| Higher | 0.80*** | 0.77 | 0.82 | 1.26*** | 1.21 | 1.31 |
| **Currently employment** |  |  |  |  |  |  |
| No | Ref |  |  | Ref |  |  |
| Yes | 1.07* | 1.02 | 1.13 | 0.92** | 0.87 | 0.98 |
| **Wealth index** |  |  |  |  |  |  |
| Poorest | Ref |  |  | Ref |  |  |
| Poorer | 1.57*** | 1.45 | 1.70 | 1.59*** | 1.46 | 1.73 |
| Middle | 2.37*** | 2.20 | 2.56 | 2.42*** | 2.23 | 2.62 |
| Richer | 3.43*** | 3.19 | 3.69 | 3.54*** | 3.26 | 3.83 |
| Richest | 4.99*** | 4.64 | 5.37 | 5.18*** | 4.78 | 5.62 |
| **Marital Status** |  |  |  |  |  |  |
| Single | Ref |  |  | Ref |  |  |
| Married | 5.16*** | 5.04 | 5.29 | 1.91*** | 1.82 | 2.00 |
| Separated/ Divorced/ Widowed | 4.74*** | 4.52 | 4.97 | 1.66*** | 1.56 | 1.76 |
| **Parity** |  |  |  |  |  |  |
| 0 | Ref |  |  | Ref |  |  |
| 1 | 3.08*** | 2.98 | 3.17 | 1.04 | 0.99 | 1.09 |
| 2 | 4.55*** | 4.43 | 4.67 | 1.18*** | 1.13 | 1.24 |
| 3 | 5.03*** | 4.88 | 5.19 | 1.29*** | 1.22 | 1.35 |
| 3+ | 4.96*** | 4.80 | 5.12 | 1.34*** | 1.27 | 1.41 |
| **Number of Household Member** |  |  |  |  |  |  |
| ≤5 | Ref |  |  | Ref |  |  |
| >5 | 0.80*** | 0.78 | 0.81 | 0.92*** | 0.90 | 0.94 |
| **Frequency of watching TV** |  |  |  |  |  |  |
| Not at all | Ref |  |  | Ref |  |  |
| Less than once a week | 1.10** | 1.04 | 1.17 | 1.00 | 0.94 | 1.07 |
| At least once a week | 1.30*** | 1.24 | 1.36 | 1.05 | 1.00 | 1.10 |
| Almost every day | 1.52*** | 1.46 | 1.58 | 1.15*** | 1.10 | 1.20 |

*NFHS: National Family Health Survey*

*CI: Confidence Interval*

*COR: Crude Odds Ratio; AOR: Adjusted Odds Ratio*

*^1^Variable with p-value less than <0.2 from unadjusted model were included into multivariable analysis*

**p-value<0.05, **p-value<0.01, *** p-value<0.001*

**Table C: Crude and Adjusted odds ratios for factors associated with overweight and obesity compared to normal weight among women of reproductive age group in India residing in rural area, NFHS 2015-16.**

| **Variable** | COR | Lower Limit | Upper Limit | AOR | Lower Limit | Upper Limit |
| --- | --- | --- | --- | --- | --- | --- |
| **Age Group (in years)** |  |  |  |  |  |  |
| 15-24 | Ref |  |  | Ref |  |  |
| 25-34 | 3.39*** | 3.32 | 3.46 | 2.38*** | 2.32 | 2.44 |
| 35-49 | 5.62*** | 5.51 | 5.73 | 4.13*** | 4.01 | 4.25 |
| **State of Residence** |  |  |  |  |  |  |
| Andaman and Nicobar islands | Ref |  |  | Ref |  |  |
| Andhra Pradesh | 0.85^1^ | 0.72 | 1.00 | 0.89 | 0.77 | 1.03 |
| Arunachal Pradesh | 0.69*** | 0.60 | 0.81 | 1.07 | 0.93 | 1.23 |
| Assam | 0.34*** | 0.29 | 0.39 | 0.57*** | 0.50 | 0.65 |
| Bihar | 0.27*** | 0.23 | 0.31 | 0.61*** | 0.53 | 0.70 |
| Chandigarh | 0.31^1^ | 0.07 | 1.32 | 0.25 | 0.06 | 1.00 |
| Chhattisgarh | 0.21*** | 0.18 | 0.24 | 0.37*** | 0.32 | 0.43 |
| Dadra and Nagar Haveli | 0.21*** | 0.14 | 0.32 | 0.39*** | 0.27 | 0.57 |
| Daman and Diu | 1.03 | 0.75 | 1.41 | 1.04 | 0.78 | 1.39 |
| Goa | 1.01 | 0.78 | 1.32 | 0.74* | 0.58 | 0.94 |
| Gujarat | 0.38*** | 0.33 | 0.44 | 0.48*** | 0.41 | 0.55 |
| Haryana | 0.62*** | 0.53 | 0.72 | 0.52*** | 0.45 | 0.60 |
| Himachal Pradesh | 0.95 | 0.81 | 1.10 | 0.78** | 0.68 | 0.90 |
| Jammu and Kashmir | 0.79** | 0.68 | 0.92 | 1.21** | 1.06 | 1.39 |
| Jharkhand | 0.17*** | 0.15 | 0.20 | 0.38*** | 0.33 | 0.44 |
| Karnataka | 0.50*** | 0.43 | 0.58 | 0.59*** | 0.52 | 0.68 |
| Kerala | 1.24** | 1.06 | 1.45 | 0.84* | 0.73 | 0.97 |
| Lakshadweep | 1.40 | 0.77 | 2.54 | 1.09 | 0.63 | 1.86 |
| Madhya Pradesh | 0.25*** | 0.21 | 0.29 | 0.43*** | 0.38 | 0.50 |
| Maharashtra | 0.39*** | 0.33 | 0.45 | 0.46*** | 0.40 | 0.52 |
| Manipur | 0.80** | 0.68 | 0.94 | 1.21* | 1.04 | 1.39 |
| Meghalaya | 0.37*** | 0.32 | 0.44 | 0.59*** | 0.50 | 0.68 |
| Mizoram | 0.47*** | 0.40 | 0.55 | 0.53*** | 0.45 | 0.62 |
| Nagaland | 0.42*** | 0.36 | 0.50 | 0.63*** | 0.54 | 0.73 |
| Delhi | 1.35 | 0.69 | 2.64 | 0.81 | 0.43 | 1.51 |
| Odisha | 0.34*** | 0.29 | 0.39 | 0.60*** | 0.52 | 0.68 |
| Puducherry | 1.27^1^ | 0.98 | 1.65 | 1.26 | 0.99 | 1.59 |
| Punjab | 1.23** | 1.06 | 1.43 | 0.95 | 0.82 | 1.09 |
| Rajasthan | 0.30*** | 0.26 | 0.34 | 0.45*** | 0.39 | 0.51 |
| Sikkim | 1.01 | 0.85 | 1.21 | 1.16 | 0.99 | 1.37 |
| Tamil Nadu | 0.86* | 0.74 | 0.99 | 0.90 | 0.79 | 1.03 |
| Tripura | 0.43*** | 0.36 | 0.51 | 0.62*** | 0.53 | 0.74 |
| Uttar Pradesh | 0.36*** | 0.31 | 0.42 | 0.70*** | 0.62 | 0.80 |
| Uttarakhand | 0.44*** | 0.37 | 0.51 | 0.51*** | 0.44 | 0.59 |
| West Bengal | 0.40*** | 0.35 | 0.47 | 0.66*** | 0.57 | 0.76 |
| Telangana | 0.54*** | 0.45 | 0.64 | 0.65*** | 0.56 | 0.76 |
| **Highest Educational Status** |  |  |  |  |  |  |
| No Formal Education | Ref |  |  | Ref |  |  |
| Primary | 1.06*** | 1.04 | 1.08 | 1.17*** | 1.15 | 1.20 |
| Secondary | 0.81*** | 0.80 | 0.83 | 1.26*** | 1.24 | 1.29 |
| Higher | 0.86*** | 0.84 | 0.89 | 1.30*** | 1.26 | 1.35 |
| **Currently employment** |  |  |  |  |  |  |
| No | Ref |  |  | Ref |  |  |
| Yes | 1.05* | 1.01 | 1.09 | 0.93*** | 0.89 | 0.97 |
| **Wealth index** |  |  |  |  |  |  |
| Poorest | Ref |  |  | Ref |  |  |
| Poorer | 1.75*** | 1.70 | 1.79 | 1.59*** | 1.55 | 1.63 |
| Middle | 2.70*** | 2.64 | 2.77 | 2.31*** | 2.24 | 2.37 |
| Richer | 4.21*** | 4.11 | 4.32 | 3.48*** | 3.37 | 3.59 |
| Richest | 6.01*** | 5.84 | 6.19 | 4.74*** | 4.57 | 4.92 |
| **Marital Status** |  |  |  |  |  |  |
| Single | Ref |  |  | Ref |  |  |
| Married | 4.53*** | 4.43 | 4.62 | 1.98*** | 1.90 | 2.05 |
| Separated/ Divorced/ Widowed | 4.35*** | 4.18 | 4.52 | 1.74*** | 1.65 | 1.83 |
| **Parity** |  |  |  |  |  |  |
| 0 | Ref |  |  | Ref |  |  |
| 1 | 2.73*** | 2.66 | 2.81 | 1.03*** | 0.99 | 1.07 |
| 2 | 3.89*** | 3.81 | 3.97 | 1.10*** | 1.06 | 1.14 |
| 3 | 3.92*** | 3.83 | 4.01 | 1.07*** | 1.03 | 1.11 |
| 3+ | 3.82*** | 3.74 | 3.91 | 1.09*** | 1.05 | 1.14 |
| **Number of Household Member** |  |  |  |  |  |  |
| ≤5 | Ref |  |  | Ref |  |  |
| >5 | 0.82*** | 0.80 | 0.83 | 0.90*** | 0.88 | 0.91 |
| **Frequency of watching TV** |  |  |  |  |  |  |
| Not at all | Ref |  |  | Ref |  |  |
| Less than once a week | 1.15*** | 1.12 | 1.19 | 1.06*** | 1.03 | 1.09 |
| At least once a week | 1.41*** | 1.37 | 1.44 | 1.11*** | 1.08 | 1.14 |
| Almost every day | 1.84*** | 1.81 | 1.88 | 1.26*** | 1.23 | 1.29 |

*NFHS: National Family Health Survey*

*CI: Confidence Interval*

*COR: Crude Odds Ratio; AOR: Adjusted Odds Ratio*

*^1^Variable with p-value less than <0.2 from unadjusted model were included into multivariable analysis*

**p-value<0.05, **p-value<0.01, *** p-value<0.001*
